# Daily Supplementation of D-ribose Shows No Therapeutic Benefits in the MHC-I Transgenic Mouse Model of Inflammatory Myositis

**DOI:** 10.1371/journal.pone.0065970

**Published:** 2013-06-13

**Authors:** William Coley, Sree Rayavarapu, Jack H. van der Meulen, Ayyappa S. Duba, Kanneboyina Nagaraju

**Affiliations:** 1 Research Center for Genetic Medicine, Children’s National Medical Center, Washington, D. C., United States of America; 2 Department of Integrative Systems Biology, George Washington University School of Medicine, Washington, D. C., United States of America; NIAID, United States of America

## Abstract

**Background:**

Current treatments for idiopathic inflammatory myopathies (collectively called myositis) focus on the suppression of an autoimmune inflammatory response within the skeletal muscle. However, it has been observed that there is a poor correlation between the successful suppression of muscle inflammation and an improvement in muscle function. Some evidence in the literature suggests that metabolic abnormalities in the skeletal muscle underlie the weakness that continues despite successful immunosuppression. We have previously shown that decreased expression of a purine nucleotide cycle enzyme, adenosine monophosphate deaminase (AMPD1), leads to muscle weakness in a mouse model of myositis and may provide a mechanistic basis for muscle weakness. One of the downstream metabolites of this pathway, D-ribose, has been reported to alleviate symptoms of myalgia in patients with a congenital loss of AMPD1. Therefore, we hypothesized that supplementing exogenous D-ribose would improve muscle function in the mouse model of myositis. We treated normal and myositis mice with daily doses of D-ribose (4 mg/kg) over a 6-week time period and assessed its effects using a battery of behavioral, functional, histological and molecular measures.

**Results:**

Treatment with D-ribose was found to have no statistically significant effects on body weight, grip strength, open field behavioral activity, maximal and specific forces of EDL, soleus muscles, or histological features. Histological and gene expression analysis indicated that muscle tissues remained inflamed despite treatment. Gene expression analysis also suggested that low levels of the ribokinase enzyme in the skeletal muscle might prevent skeletal muscle tissue from effectively utilizing D-ribose.

**Conclusions:**

Treatment with daily oral doses of D-ribose showed no significant effect on either disease progression or muscle function in the mouse model of myositis.

## Introduction

Idiopathic inflammatory myopathies are characterized by symmetrical muscle weakness and easy fatigability. Examinations of patients’ skeletal muscle tissue typically reveal signs of fiber size variation, muscle fiber degeneration, and autoimmune inflammation. In humans, the most commonly diagnosed idiopathic inflammatory myopathies are polymyositis, dermatomyositis, and inclusion body myositis, commonly referred to as myositis. Since muscle inflammation is a predominant feature of histological disease, these diseases are treated with immunosuppressants such as prednisone or methotrexate. These therapies successfully modulate the disease activity and show clinical benefits in a majority of patients. However, patients are expected to neither fully recover muscle function nor be cured of the disease by current therapies. The reasons for the lack of full response to immunosuppressive therapies are unknown. There is evidence in the literature to suggest that metabolic abnormalities in the skeletal muscle of myositis patients may contribute to muscle weakness independent of the inflammation and damage caused by autoimmune response [1], [2], [3]. In patients, it was previously observed that muscle weakness is associated with an acquired deficiency of the adenosine monophosphate deaminase (AMPD1) enzyme [4], [5]. We recently confirmed these findings in the MHC class I transgenic mouse model of myositis [6]. “Similarly, patients with a congenital loss of AMPD1 enzyme activity are also reported to experience symptoms of muscle weakness and fatigue [7], [8]. There are case reports suggesting that oral ribose administration reduced exercise-related symptoms in an AMPD1-deficient patient, as well as cases that have shown no benefit after taking ribose [9], [10], [11]. In order to examine the therapeutic potential of D-ribose more thoroughly, we decided to test the therapeutic efficacy of D-ribose in the mouse model of myositis. This mouse model has proven to very closely mimic some features of human polymyositis [12]. Furthermore, novel observations made using this disease model were later confirmed to be present in human patients [13], [14]. We consider this is an appropriate mouse model to evaluate potential therapies.

We have now demonstrated that there is a deficiency of AMPD1 as well as hypoxanthine and other AMP breakdown products in myositis mice, but treatment of these mice with daily oral doses of D-ribose showed no beneficial effects. These results in our mouse disease model are consistent with other reports indicating that D-ribose has no effect on muscle performance in healthy patients or in patients with other metabolic diseases [15], [16], [17]. We also propose that low expression levels of the enzyme ribokinase may contribute to inefficient utilization of D-ribose in skeletal muscle.

## Materials and Methods

### Animals

All mice were handled according the local guidelines established by the Institutional Animal Care and Use Committee (IACUC) of the Washington D.C. VA Medical Center, and all procedures were carried out under the approved animal protocol (#00993). Animals were housed at room temperature with a 12–12-h light-dark cycle. Genotyping was carried out at 3–4 weeks as previously described [18]. The mouse model of myositis used in this study is known as the MHC-I overexpression transgenic model and has been described previously in multiple publications [6], [12], [19]. In brief, the mouse model of myositis utilizes a double-transgenic mouse, in which the T gene (an MCK promoter-driven *tet*-Off transcription factor) forces the expression of the H gene (a TRE-driven H-2Kb MHC class I molecule). Double-transgenic animals are labeled “HT” and spontaneously develop myositis after doxycycline withdrawal. Single-transgenic animals are referred to as “H” mice and do not develop myositis regardless of doxycycline administration. For all animals, doxycycline was withdrawn from the water supply at 5 weeks of age. The constitutive forced expression of MHC class I results in a chronic ER stress response in the muscle. In this model, female mice show the first sign of pathology at about 8 weeks of age, when a drop in AMPD1 enzyme activity and a drop in fast twitch muscle strength can be measured [4]. HT mouse body weight gain will typically plateau around 10 weeks of age, and may drop over the following months. The infiltration of lymphocytes is not typically seen until the animals reach 13 weeks of age or older. AMPD1 enzyme activity and muscle function continue to decrease up to 16 weeks of age, and may continue to drop over the following months. Measurable decreases in grip strength and open field activity can be seen at 16 weeks of age. For ethical reasons, mice are typically euthanized by 20 weeks of age. Measurable decreases in grip strength and voluntary movement can be seen as early as 16 weeks of age. Mice younger than 20 weeks have no difficulty feeding and drinking *ad libitum*.

### Treatment Schedule

For controls, single-transgenic age- and sex-matched littermates were used. All mice were left untreated until they reached 10 weeks of age, at which point they were randomly assigned to either a control group or treatment group. D-ribose (Sigma-Aldrich R9629) was dissolved in double-distilled water and prepared fresh weekly for treatments. A measured volume of D-ribose was delivered by a syringe and voluntarily ingested by the mice. The dosage given was 4 mg/kg daily between 10∶00am and 12∶00pm. The body weight of each mouse was measured weekly. Symptoms of myositis were readily apparent by 15 weeks of age. Since the disease progresses at different rates in male mice versus female mice, only female mice were utilized for this trial. Functional, behavioral and histological data was collected and analyzed in a blinded fashion. At the end of the experiments, the mice were killed by CO_2_ inhalation followed by cervical dislocation, according to IACUC guidelines. Muscle tissue was immediately dissected and flash-frozen in isopentane pre-chilled with liquid nitrogen and stored at −80°C.

### Histology

Frozen muscle tissues were mounted in Optimal Cutting Temperature compound (Tissue-Tek) and cut on a microtome to produce 8 µm-thick cross-sections. Sections were allowed to air dry for 5 min., and then stained with filtered 0.1% Mayer’s Hematoxylin (Sigma-Aldrich) for 10 min. Stained sections were then rinsed in running ddH_2_0 for 5 minutes and then dipped in 0.5% Eosin 12 times. Stained slides were then washed in ddH_2_0 to remove excess Eosin and then progressively dipped 10 times each in 50% ethanol, 70% ethanol, 95% ethanol, and 100% ethanol. Slides were then dipped in Xylene, cleaned with a Kimwipe, and mounted with Permount (Fisher Scientific) and coverslips. For CD3 staining in muscle sections, 8 micron-thick sections were first allowed to air dry for 5 minutes, then incubated in blocking solution (5% BSA and 10% Normal Sheep serum in PBS) for 1 hour at room temperature, washed in PBS, then incubated with primary anti-CD3 antibody ([ab16669, Abcam] diluted 1∶50 in PBS+0.5% BSA) overnight at 4°C. Slides were then washes in PBS before incubation with anti-Rabbit HRP secondary ([P0448, Dako] diluted 1∶200 in PBS+0.5% BSA) for 1 hour at room temp, then developed using a DAB substrate Kit (SK-4100, Vector) for 9 minutes at room temp. Stained slides were rinsed in ultrapurified water, counterstained with Hematoxylin, and rinsed again for 10 min. Slides were allowed to dehydrate overnight and then mounted with Permount (Fisher Scientific) and coverslips. Images were captured using a Nikon Eclipse E800 microscope with an attached Spot RT Slider digital camera. Counting was performed in a blinded fashion, where a focus was defined as either 3 or more CD3+ cells adjacent to each other.

### Functional and Behavioral Activities

#### Grip strength test

Grip strength in both thoracic and pelvic limbs was assessed using a grip strength meter consisting of a horizontal thoracic limb mesh and an angled pelvic limb mesh (Columbus Instruments, Columbus, OH) according to a previously published protocol [20]. Five successful thoracic limb and pelvic limb strength measurements were recorded within 2 min. The maximum values for each day over a 5-day period were averaged and normalized to body weight and expressed as KGF/kg unit. All grip strength measurements were begun when mice were 15 weeks of age and concluded prior to animal sacrifice at 16 weeks of age.

#### Behavioral activity

Activity in an open field was measured using an open-field Digi-Scan apparatus (Omnitech Electronics, Columbus, OH) as described previously [20]. The data were collected every 10 min over a 1-hr period each day for 4 consecutive days. The results were calculated as means ± standard error for all recordings. The recorded quantitative measures of activity were horizontal activity, vertical activity, and total distance. Total distance is measured in centimeters, while horizontal activity and vertical activity are measured as the number of beam breaks (arbitrary units). All Digi-Scan measurements were begun when mice were 15 weeks of age and concluded prior to animal sacrifice at 16 weeks of age.

### Force Contractions on Isolated Skeletal Muscle

Force contraction experiments were conducted on the extensor digitorum longus (EDL) and soleus muscles of the right pelvic limbs of mice. The mouse was anesthetized with an intraperitoneal injection containing ketamine (100 mg/kg) and xylazine (10 mg/kg). The muscles were isolated, and 6–0 silk sutures were tied securely to the distal and proximal tendons. Each muscle was then carefully removed from the mouse and placed vertically in a bath containing buffered mammalian Ringer’s solution (137 mM NaCl, 24 mM NaHCO_3_, 11 mM glucose, 5 mM KCL, 2 mM CaCl_2_, 1 mM MgSO_4_, 1 mM NaH_2_PO_4_ and 0.025 mM turbocurarine chloride) maintained at 25°C and bubbled with 95% O_2_-5% CO_2_ to stabilize the pH at 7.4. The distal tendon of the muscle was tied securely to the lever arm of a servomotor/force transducer (model 305B, Aurora Scientific), and the proximal tendon was tied to a stationary post in the bath. After removal of the muscle, the mouse was euthanized by gassing with CO_2_ according to IACUC guidelines. The muscle was stimulated between two stainless steel plate electrodes. The voltage of single 0.2-msec square stimulation pulses was increased until supramaximal stimulation of the muscle was achieved, and the muscle length was then adjusted to the length that resulted in maximal twitch force (i.e., optimal length for force generation). With the muscle held at optimal length, the force developed during trains of stimulation pulses was recorded, and stimulation frequency was increased until the maximal isometric tetanic force was achieved. For the EDL muscle, 300-ms trains of pulses were used, and a stimulus frequency of ∼220 Hz was typically needed to achieve the maximum isometric force. Maximal isometric force for the soleus muscle was achieved at a frequency of 120 Hz with 1000-ms trains. The muscle length was measured with calipers, and the optimal fiber length was calculated by multiplying the optimal muscle length by a constant of 0.45, an established fiber length/muscle length ratio for EDL muscle, and 0.71 for the soleus muscle. The muscle mass was weighed after removal of the muscle from the bath. The muscle-specific force, a measure of the intrinsic force generation of the muscle, was calculated according the following equation: specific force = maximal isometric force/(muscle mass _*_ (density of muscle tissue _*_ fiber length) ^−1^). The muscle tissue density was 1.056 kg/L.

### Quantification of Metabolites

#### Quantification of hypoxanthine

Frozen skeletal muscle (quadriceps) was homogenized in a liquid N_2_-chilled ceramic mortar and pestle, and then lysed by repeated freeze-thaw cycles (x3) in RIPA buffer (Sigma-Aldrich). The concentration of soluble protein was determined, and the cleared lysates were stored at −80°C. The concentration of hypoxanthine in 50 ml of cleared lysate was then determined with an Amplex Red Xanthine/Xanthine Oxidase Kit (Life Technologies) according to the manufacturer’s protocol. The levels of hypoxanthine for each sample were then normalized using the protein concentration of each sample.

#### Quantification of inosine monophosphate

Intramuscular levels of IMP were determined by triple-quad mass spectrometry. In brief, frozen skeletal muscle (quadriceps) was homogenized in a liquid N_2_-chilled ceramic mortar and pestle and then resuspended in 250 µl of 50% methanol solution to precipitate protein. The IMP internal standard was also resuspended in 50% methanol. Quantities for IMP were acquired in triplicate runs on a Xevo TQ mass spectrometer (Waters). Serial dilutions of the IMP internal standard (0.1–1275 pmoles/µl) were prepared, and a standard curve made from the measurements after being acquired three times. Two transitions were selected to quantify the concentration in each sample. Target Lynx software (Waters) was used to quantify the concentration of IMP the in the samples, and the quantity of each sample was normalized to the protein concentration of each sample, as determined by a Bradford protein concentration assay.

#### Quantitative QRT-PCR analysis

To isolate RNA for QRT-PCR analysis, frozen EDL and soleus muscle tissues were diced with a sterile razor and homogenized in Trizol (Life Technologies) using a Kontes pestle. After isolation according to the manufacturer’s instructions, RNA was quantified on a Nanodrop N1000 spectrophotometer. A total of 800 ng of RNA was used to produce cDNA using a Promega Reverse Transcription System kit. Primers were designed for mouse AMPD1, ADSL, ADSSL1, NT5C2, PNP, CKM, HK2, RBKS, and GAPDH using Primer3 v0.4.0 software [21]. The sequences of the primers used were: AMPD1 forward, TATCA GCATG CAGAG CCTCG CTTA; AMPD1 reverse, TGTGG CAGGA AATTC TTGGA TCGG; ADSSL1 forward, AGACT CTCCC AGGAT GGAAC; ADSSL1 reverse, GTTGC TGGCA ATCCT TAGAA; ADSL forward, TACTT CAGCC CCATC CACTC; ADSL reverse, TCACT GTAAC CGGGT TCTCC; NT5C2 forward, CCCAT TCAGC TACCT CTTCA; NT5C2 reverse, ATGGC AGTGT GTGAT CTCCT; PNP forward, GGCTT CTGCA ACACA CTGAA; PNP reverse, TTCAG CAATC CAAAC ACCAG; CKM forward, GATTC TCACT CGCCT TCGTC; CKM reverse, GCCCT TTTCC AGCTT CTTCT; HK2 forward, AGAAC CGTGG ACTGG ACAAC, HK2 reverse, GCCAG ATCTC TCACC GTCTC; RBKS forward, AAGAA GGCAG CCAGT GTCAT; RBKS reverse GAGCT GGGTT GAACA AGGTT; CCL5 forward, GTGCC CACGT CAAGG AGTAT; CCL5 reverse, CCCAC TTCTT CTCTG GGTTG; IL-1β forward, GGGCC TCAAA GGAAA GAATC; IL-1β reverse, TACCA GTTGG GGAAC TCTGC; IFIT2 forward, CGCTT TGACA CAGCA GACAG; IFIT2 reverse, TGCAG TGCTG CCTCA TTTAG; MXB forward, AGGAG GAAGC TGAGG AGGAG; MXB reverse, ACTGG ATGAT CAAGG GAACG; CD3e forward, ATCAC TCTGG GCTTG CTGAT; CD3e reverse, GTCCA CAGAA GGCGA TGTCT; CD19 forward, GTTGG CAGGA TGATG GACTT; CD19 reverse, TCCCA TGCTG GTTCT AGGTC; EMR1 forward; CCATT GCCCA GATTT TCATC, EMR1 reverse, GGTCA GTCTT CCTGG TGAGG; GAPDH forward, CGTCC CGTAG ACAAA ATGGT; GAPDH reverse, GAATT TGCCG TGAGT GGAGT. All primers were verified to produce single, specific amplicons of the correct size before being used for QRT-PCR. All QRT-PCR reactions were prepared according to the manufacturer’s protocol using a hot-start SYBR green premade mix (NEB F-410) and measured on an ABI HT7900 thermal cycler. Relative gene expression was calculated using the ΔΔCt method, with GAPDH as the internal reference gene.

## Results

### An Acquired Loss of AMPD1 is Correlated with a Loss of Downstream Metabolites

**Figure 1:**
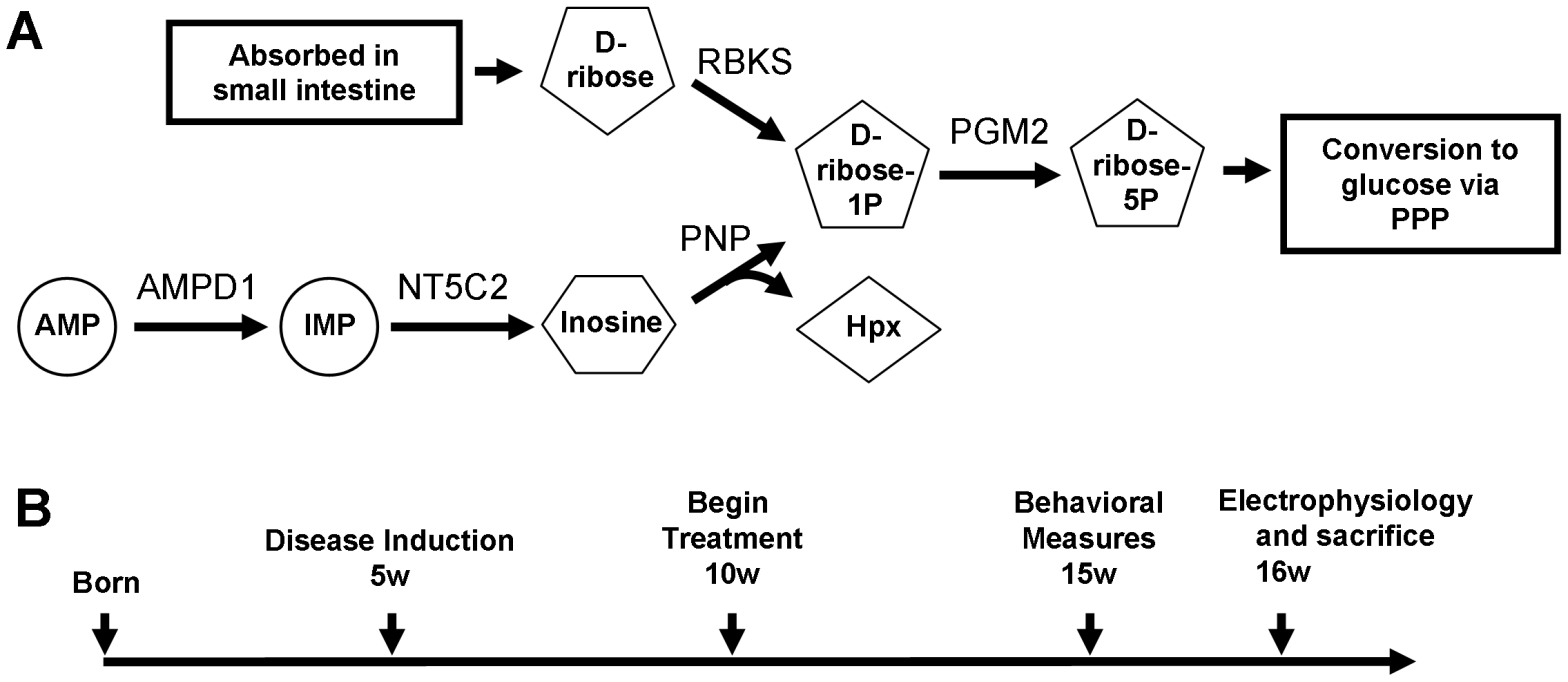
AMP catabolism can generate free D-ribose. The catabolism of AMP into hypoxanthine (Hpx) and ribose is illustrated, along with the utilization of ingested D-ribose (*A*). The treatment schedule of mice is depicted (*B*). Mice received a total of 6 weeks of daily oral D-ribose supplements. Behavioral assays require 5 days to conduct, and were therefore carried out one week prior to sacrificing the animals and performing electrophysiology. Abbreviations: IMP: inosine monophosphate, Hpx: hypoxanthine, D-ribose-1P: D-ribose-1-phosphate, D-ribose-5P: D-ribose-5-phosphate.

**Figure 2:**
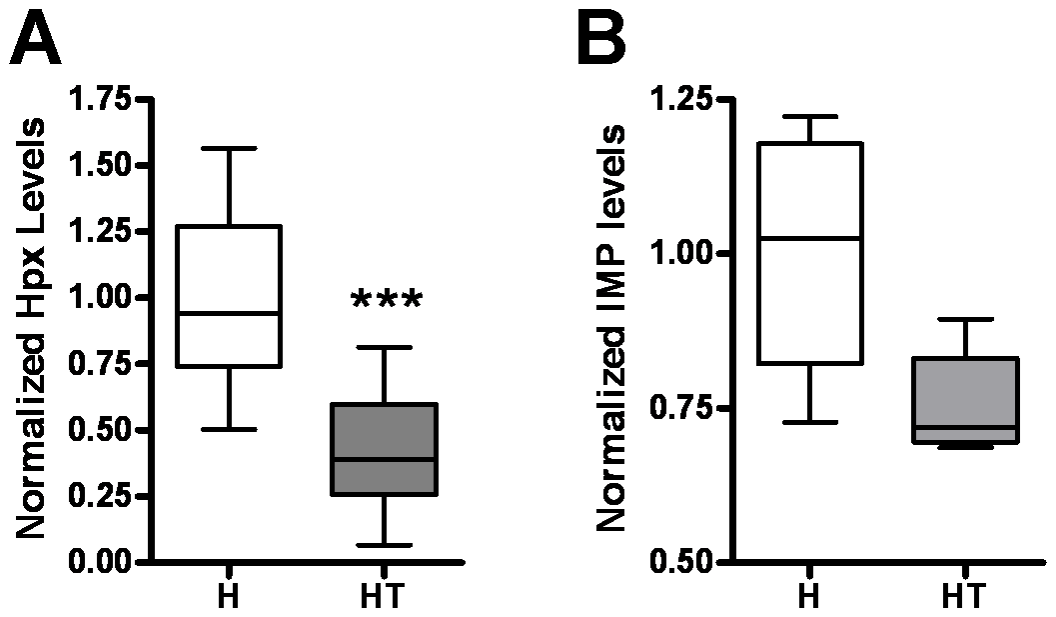
Mice with myositis are deficient for the breakdown metabolites of AMP. Hypoxanthine levels in mice with myositis (HT mice, n = 10) were significantly lower than in healthy (H mice, n = 10) littermates (*A*). IMP levels in HT mice were also lower in HT mice than in healthy controls (*B*). All mice involved in metabolite assays were 16-week-old females. Metabolite levels were measured in lysates from quadriceps muscle tissue.

Metabolic pathway that links the catabolism of AMP and the production of D-ribose within skeletal muscle are depicted in Figure 1. To verify that this pathway was affected by the acquired deficiency of AMPD1, we quantified the relative abundance of the metabolites inosine monophosphate (IMP) and hypoxanthine (Fig. 2). Quadriceps muscles from 16-week-old H and HT mice were dissected, flash-frozen, and then homogenized to generate skeletal muscle lysates. IMP is the immediate downstream product of AMPD1, and we found that IMP levels in the myositis mice were 24.6% lower than those in healthy controls (Fig. 2B). A more significant deficit was seen in hypoxanthine levels in HT mice (58.2% lower) (7.17 µM, n = 10) when compared to healthy controls (17.19 µM, n = 10). Hypoxanthine is produced simultaneously with ribose-1P at a 1∶1 ratio and therefore serves as an indicator of the amount of free ribose-1P that is produced within the skeletal muscle fibers. These data indicate that supplementing D-ribose could potentially compensate for an acquired deficiency in AMPD1 activity.

### Effects of D-ribose Treatment on Behavioral Activity

**Figure 3:**
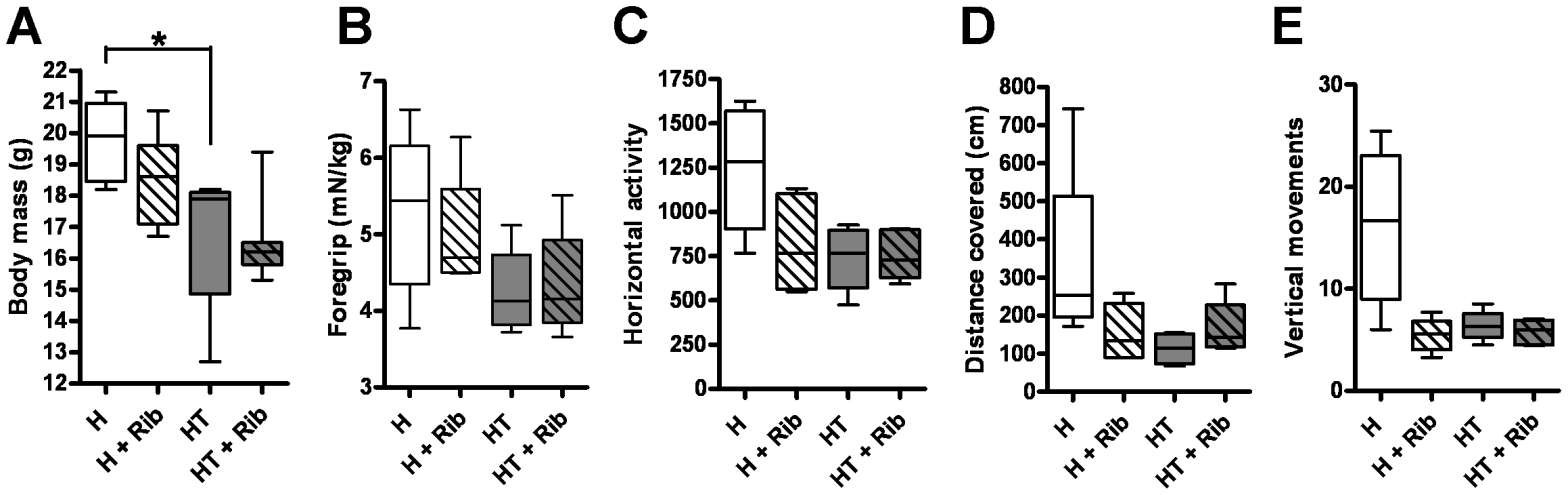
Treatment with daily oral D-ribose did not improve mouse body weight, grip strength and open field behavioral activity. Data from four treatment groups are shown: Healthy untreated mice (H, n = 8), healthy mice given daily oral D-ribose (H+Rib, n = 7), untreated myositis mice (HT, n = 6), and myositis mice given daily oral D-ribose (HT +Rib, n = 8). The body weight for all animals was measured at 16 weeks of age (*A*). Thoracic limb grip strength for all animals was measured over 5 days and normalized to body weight (*B*). Mouse voluntary movement in an open field was measured as movement in an hour averaged over 5 days. Horizontal motion measured how many times an infrared beam was broken in the open field (*C*). Distance traveled was measured by how many centimeters each mouse moved (*D*). Vertical movements indicate the number of times each mouse stood upright (*E*). Overall, the onset of myositis resulted in a significant loss of body weight, while grip strength measurement did not discriminate well between groups. Treatment with ribose showed no beneficial effect on any of the measured parameters.

Mice were treated with oral supplements of D-ribose according to the schedule depicted in Fig. 1B. Doxycycline was withdrawn at 5 weeks of age to initiate the onset of myositis in HT mice, while H mice remained healthy. Treatment with D-ribose began when mice were 10 weeks old and continued until mice were 16 weeks old. This treatment schedule was chosen to reflect the reality that the diagnosis and treatment of patients cannot begin until the disease has already set in. Four groups were evaluated in this trial: untreated healthy mice (H), healthy mice given daily doses of oral D-ribose (H+Rib), untreated myositis mice (HT), and myositis mice treated with daily doses of oral D-ribose (HT+Rib). The onset of myositis resulted in a significant decrease in body weight (14.2±0.5% decrease) in HT mice (Fig. 3A). Treatment with D-ribose resulted in no significant change in body weight but showed a trend towards a decrease in weights. Untreated HT mice showed reduced thoracic limb grip strength when compared to untreated H mice, a 19.2±0.1% decrease. Treatment with D-ribose did not alter thoracic limb grip strength in either treatment group (Fig. 3B).

We measured open-field behavioral activity using a Digi-scan chamber and found that HT mice showed reduced total distance, horizontal activity, and vertical movements when compared to H mice. Treatment of H mice with ribose resulted in decreased horizontal activity (35.2±0.1% decrease, Fig. 3C), total distance (60.6±21.7% decrease, Fig. 3D), and vertical movements (64.7±34.1% decrease, Fig. 3E) when compared to untreated H mice. Treatment with ribose did not significantly alter the open-field activity of the HT mice (Fig. 3C–E).

### D-ribose Treatments did not Improve Body Weight Gain

**Figure 4:**
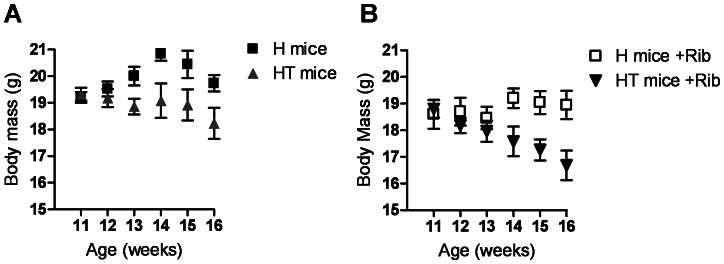
Treatment with oral doses of D-ribose does not improve body mass over time. Body weights for mice were recorded starting at the time of D-ribose administration. Mice were sacrificed at 16 weeks of age. The gap in bodyweight between healthy controls and untreated diseased mice widened over time (*A*). Treatment with D-ribose had no apparent failed to prevent weight loss in HT animals (*B*).

Mice were treated with oral supplements of D-ribose beginning when the animals were 10 weeks old, and continued until the animals were sacrificed at 16 weeks. Mice were weighed weekly during this time, and the bodyweights of the treatment groups are presented in Figure 4. Untreated Healthy mice (H) show normal weight gain while untreated myositis mice (HT) show a progressive weight loss (Fig. 4A). Treatment with D-ribose did not ameliorate weight loss in diseased mice (Fig. 4B). A two-way ANOVA statistical analysis confirmed that treatment resulted in no significant differences between either healthy (H vs. H+Rib) animals, or myositis (HT vs. HT+Rib) mice.

### Effect of D-ribose on Skeletal Muscle Histology and Markers of Inflammation

**Figure 5:**
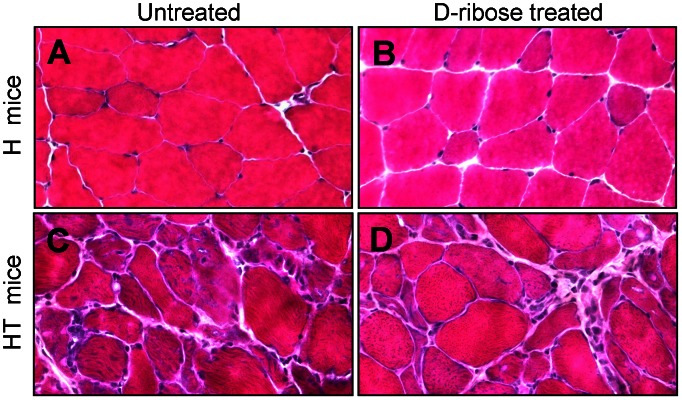
Histological analysis of mouse skeletal muscle showed no difference between untreated and treated animals. After mice were sacrificed at 16 weeks of age, quadriceps tissue was sectioned and stained with H&E. Untreated healthy mice (H) showed normal muscle histology (*A*). Healthy mice treated with daily oral D-ribose (H+Rib) also showed normal muscle histology (*B*). Untreated myositis mice (HT) showed variable muscle fiber diameter consistent with myositis (*C*). Myositis mice treated with daily oral D-ribose (HT+Rib) showed no apparent improvement at the histological level (*D*).

For hematoxylin and eosin (H&E) staining, quadriceps muscles from H and HT mice were dissected, flash-frozen, sectioned, and stained with H&E (Figures 5A–D). The images shown are representative of the histological appearance seen in each treatment group. The untreated H mice (Fig. 5A) possessed normal muscle histology, while untreated HT myositis mice (Fig. 5C) showed inflammation and abnormal variations in muscle fiber size. In order to visualize infiltrating T-cells, we performed immunohistochemical staining for CD3 (Supplemental Fig. S1). We found multiple foci of T-cell infiltrates in the muscle sections of HT mice. No CD3-positive cells were detected in sections from healthy mice. Treatment with D-ribose did not reduce the number of inflammatory foci present observed in HT mice (Fig. S1E).

**Figure 6:**
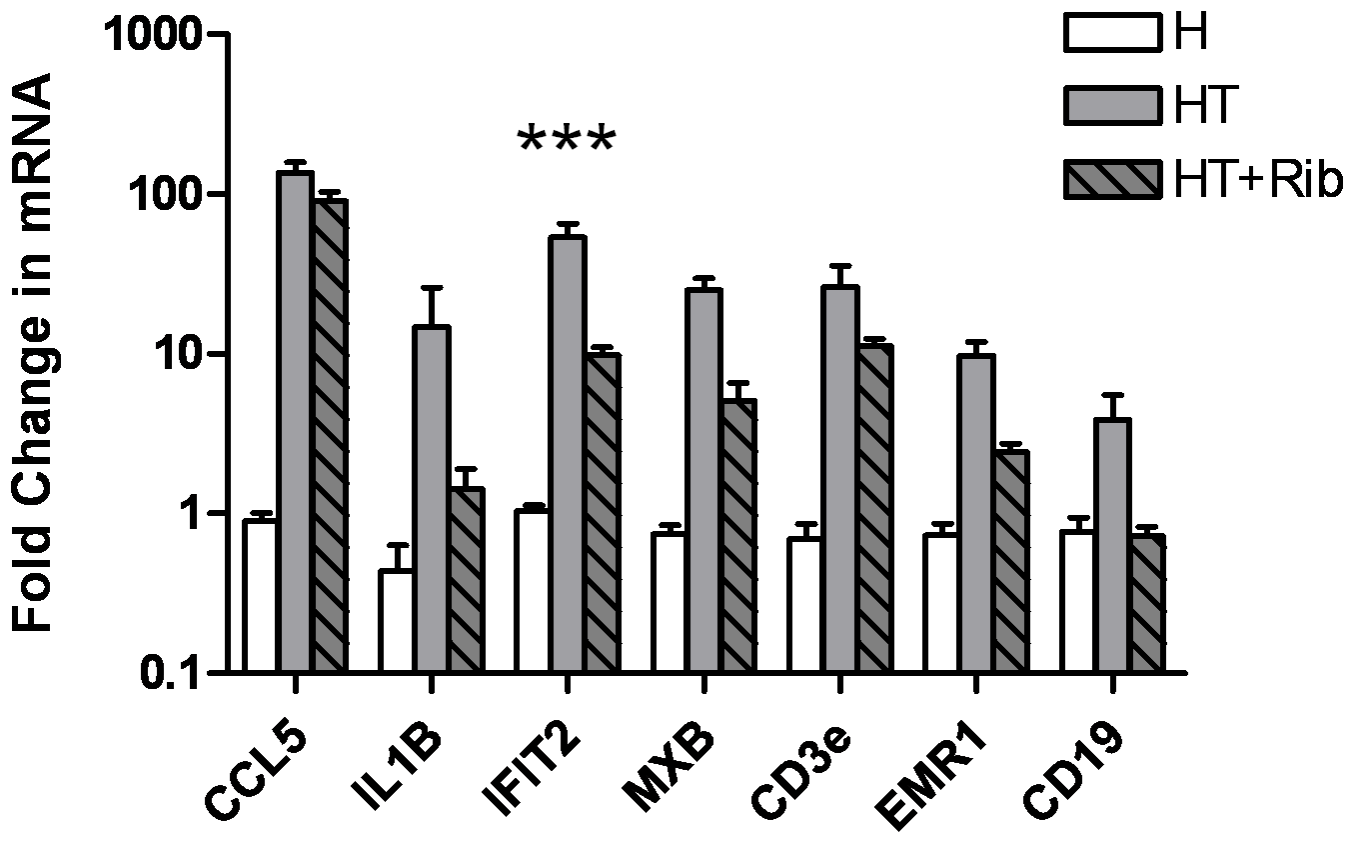
Treatment with ribose did not significantly reduce expression of inflammatory markers. After mice were sacrificed, RNA was isolated from the quadriceps muscle tissue and used to determine the expression of several markers of inflammation. The expression of each gene was normalized to the level of GAPDH mRNA. With the exception of IFIT2, there was no statistically significant reduction in the expression of these genes related to inflammation when untreated HT and ribose treated HT mice were compared.

While histology is useful for identifying infiltrations and muscle damage, histological sections can be misleading because inflammation is patchy in muscle sections. To better quantify the amount of inflammation in the skeletal muscle tissue of treated mice, we used QRT-PCR to examine the expression of several genes related to inflammation. Fig. 6 shows the QRT-PCR results from the quadriceps muscle tissue of healthy mice (H), untreated myositis mice (HT), and treated myositis mice (HT+Rib). Transcripts for the cytokines CCL5 (also known as RANTES) and IL-1β were both found to be significantly upregulated in HT mice relative to untreated control H mice. Treatment with D-ribose failed to significantly reduce the expression of these genes. The expression of the IFIT2 and MXB genes is upregulated by Type 1 interferons, and both were significantly upregulated in HT mice when compared to untreated control H mice. Treatment with ribose resulted in a significant decrease in IFIT2 transcripts, but not in MXB transcripts. Expression of CD3e, CD19, and EMR1 (also known as F4/80) mRNA indicated the presence of T cells, B cells, and macrophages, respectively. The CD3e and EMR1 transcripts were significantly upregulated in HT mice when compared to healthy mice, but not significantly altered by treatment with D-ribose. In the case of CD19, there were no statistically significant differences among the three groups. From these data, we concluded that treatment of HT mice with ribose did not affect the inflammation.

### Effect of D-ribose on Muscle Function in the EDL and Soleus Muscle

**Figure 7:**
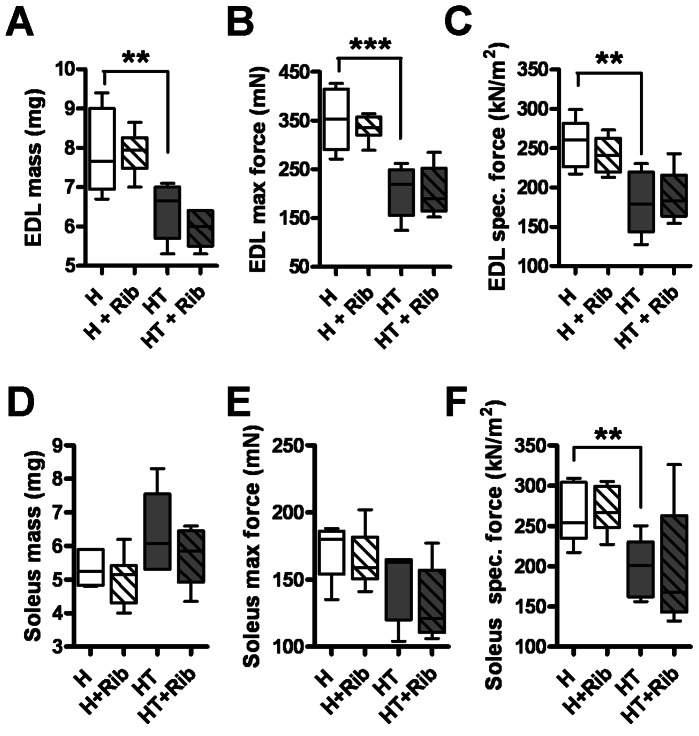
Muscle force contraction analysis of the EDL and soleus showed no differences between treated and untreated groups. The EDL (*A–C*) and soleus (*D–F*) muscles were dissected out of anesthetized 16-week-old mice and subjected to force contraction analysis. The wet mass of both muscles was measured following force contraction analysis (*A,D*). The maximal force exerted by each muscle was measured (*B,E*). The specific force that each muscle was able to produce was measured (*C,F*). Specific force was calculated using the maximal force and cross-sectional area of each muscle. Treatment with ribose showed no significant effect on the performance of either the EDL or soleus muscle.

Type 1 (slow-twitch) and Type 2 (fast-twitch) muscle fibers are differentially affected in myositis patients and in the mouse model. Therefore, we assessed muscle function in both Type 1 (soleus) and Type 2 (EDL) muscles. The EDL muscle mass was significantly lower (18.01±0.1% decrease) in HT mice than in control H mice, but the mass of the soleus was not significantly reduced (compare Fig. 7A and 6D). Similarly, the maximum force produced from the EDL was significantly lower in myositis mice (29.45±0.1% decrease) when compared to H mice, but the maximal force generated by the soleus was not significantly diminished (compare Fig. 7B and 6E). These differences suggest that Type 2 muscle fibers are more severely affected at 16 weeks of age in this mouse model. The specific force generated by the EDL and soleus muscles was found to be significantly lower in diseased mice than in healthy controls (−41.04±0.1% in the EDL and −25.17±0.1 in the soleus). Treatment with D-ribose showed no significant improvement in specific force for either the EDL or the soleus in this mouse model.

**Figure 8:**
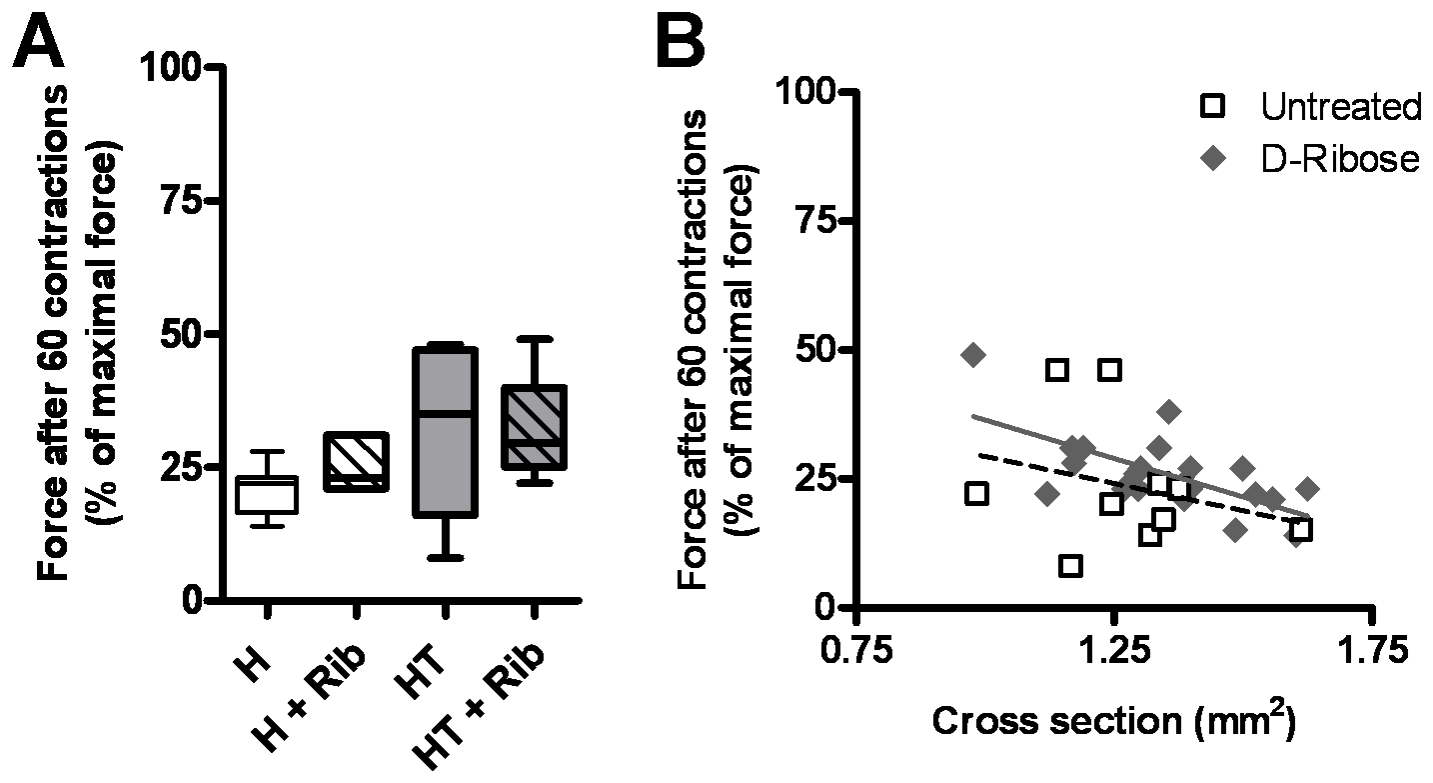
Treatment with D-ribose showed negligible effects on the fatigability of the EDL muscle. The EDL muscle was dissected out of anesthetized 16-week-old mice and subjected to fatigue resistance analysis. Fatigability was measured for all four treatment groups (*A*). The relationship between fatigue resistance and EDL fiber cross sectional area was graphed on a scatter plot (*B*). Linear regression was used to formulate the trend lines. Treatment with daily oral D-ribose showed no significant improvement in fatigue resistance in treated mice when compared to untreated mice. The two approximately parallel trend lines show that relationship between fiber size and fatigue resistance may account for the apparent increase in fatigue resistance seen in HT animals.

The EDL muscles were also tested for fatigue resistance after completing the force contraction analysis. The soleus was not tested because slow-twitch muscles are considered resistant to fatigue. Fatigue resistance is measured as a ratio between the maximal force production and the force production remaining after 60 consecutive contractions. The EDL muscles from HT myositis mice showed fatigue resistance when compared to H mice. The fatigue resistance seen in HT mice has also previously been observed by other investigators in this mouse model [22]. Treatment with oral D-ribose did not have any significant effect on the fatigue resistance of the EDL in either treatment group (Fig. 8A). The increased fatigue resistance seen in HT animals could be related to the size of the fiber sand the rate at which metabolites such as lactic acid and H^+^ can diffuse out of the muscle during the 60 consecutive contractions. This is a particular concern here, since Fig. 7A shows a significant drop in the average muscle mass between H and HT mice. This experimental artifact is visible in Fig. 8B, in which we plotted EDL muscle fatigue resistance against EDL cross-sectional area for all untreated (H and HT) and all D-ribose treated mice (H+Rib and HT+Rib). The linear regressions for the two groups formed nearly parallel lines, and while the y-intercept for the D-ribose-treated animals was larger than the y-intercept for untreated animals, the results for the two groups were not statistically different.

### Expression of RBKS in Skeletal Muscle

**Figure 9:**
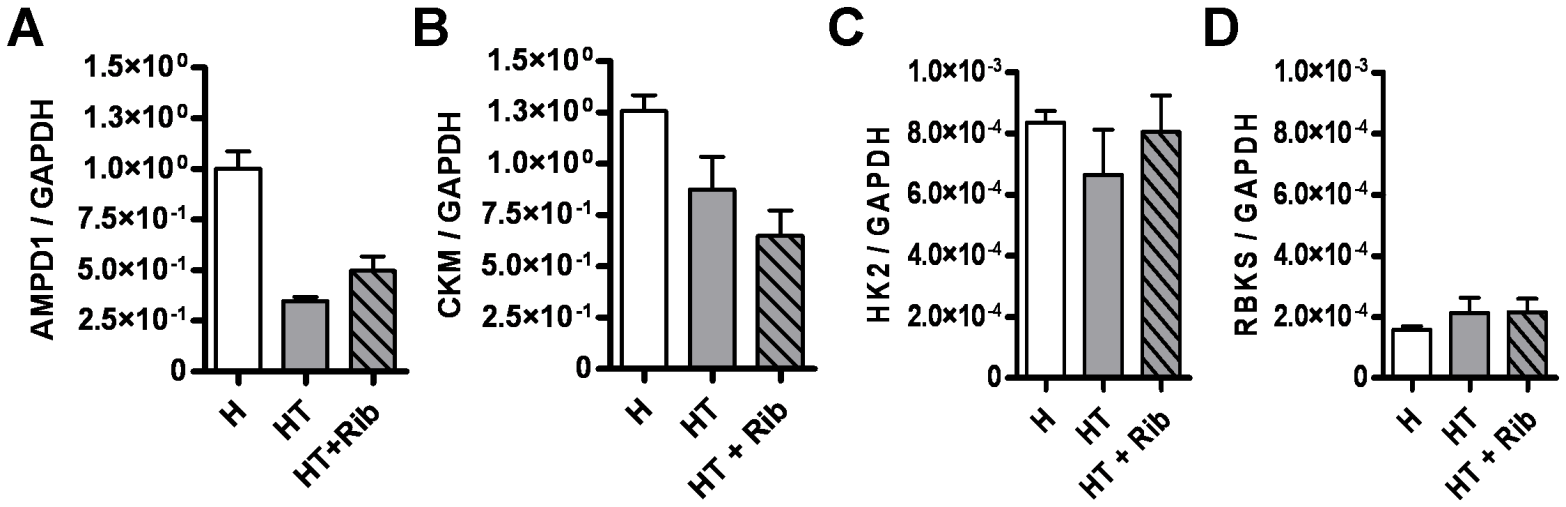
Quantitative RT-PCR analysis suggests that a lack of RBKS prevents the utilization of D-ribose. After mice were sacrificed, RNA was isolated from the quadriceps muscle tissue and used to determine the expression of several genes. The expression of each gene was normalized to the level of GAPDH mRNA. QRT-PCR was performed to measure the expression of AMPD1 *(A)* and muscle creatine kinase (CKM) in the quadriceps muscle of mice (*B*). Results for the expression of ribokinase (RBKS) in the quadriceps muscle in mice are shown (*C*), as are results for hexokinase (HK2) in mice (*D*). HT mice showed an acquired deficiency of AMPD1 expression that was not significantly affected by daily oral D-ribose treatments. QRT-PCR analysis also revealed that RBKS was 5.53-fold less abundant than hexokinase in both healthy and diseased mice.

We quantified the expression of each of the enzymes shown in the pathway depicted in Fig. 1, as well that of as other genes important for muscle metabolism. The genes for both AMPD1 and muscle creatine kinase (CKM) are known to be downregulated in humans and mice with myositis. As mentioned previously, AMPD1 is believed to control AMP levels in skeletal muscle, while CKM is essential for regulating levels of phosphocreatine. The data obtained for AMPD1 (Fig. 9A) and CKM (Fig. 9B) show that both genes are downregulated in untreated HT mice when compared to untreated H mice. Treatment with a daily dose of oral D-ribose had no significant effect on the expression of these genes when untreated and treated HT mice were compared. QRT-PCR analyses of the expression of NT5C2, PNP, and PGM2 showed that each of these genes was expressed in the skeletal muscle, but no significant differences in gene expression were observed between any of the treatment groups (data not shown). However, QRT-PCR analysis of RBKS and hexokinase 2 (HK2) revealed that the basal expression of RBKS was only 18.9% that of HK2 expression. The low expression of RBKS suggests that mouse muscle cells may not effectively retain and metabolize ingested D-ribose.

## Discussion

In this work, we used a mouse model of myositis to investigate the therapeutic potential of daily oral D-ribose supplements for the treatment of myositis. Treatment with D-ribose did not significantly affect body weight, grip strength, open-field behavioral activity, muscle histology, or expression of inflammatory markers in mouse skeletal muscle. The performance of the EDL and soleus muscles was not affected by treatment with D-ribose with regard to any of the measured parameters (maximal force, specific force, and fatigue resistance). Gene expression analysis suggested that a low basal expression of the ribokinase enzyme in mouse skeletal muscle tissues, which might prevent the effective utilization of, ingested D-ribose.

Myositis is an autoimmune disease of skeletal muscle, and patients are typically treated with immunosuppressive drugs (e.g. prednisone, methotrexate), but metabolic abnormalities are not addressed. However, it has been observed that there is a poor correlation between the successful suppression of inflammation and recovery of muscle function [1], [3], indicating that non-immune metabolic defects are potential therapeutic targets in this disease. This work was originally proposed on the basis of a combination of previously published findings. First, it has been observed that both patients and mice with myositis acquire a deficiency of the muscle specific enzyme AMPD1 [4], [6]. Second, there are published case reports describing the successful treatment of muscle weakness and fatigue with D-ribose in patients with congenital deficiency of AMPD1 enzyme activity [9], [10], [11]. Finally, an examination of the metabolites within the muscle tissue of myositis mice revealed a deficiency in metabolites related to the internal production of D-ribose. It should be noted that while D-ribose is not known to have any effect on healthy individuals, who are not expected to have an AMPD1 deficiency. Taken together, these observations suggested that in mice with myositis, supplementing the mice with D-ribose could reverse the symptoms of muscle weakness and fatigue. Despite the lack of data concerning treatment for myositis, the lay community has touted D-ribose as a treatment for both myositis and fibromyalgia. Since patients with myositis are expected to have acquired deficiency in AMPD1 enzyme activity, treatment with D-ribose remained an untested potential therapeutic option.

Before we undertook this trial, we first identified a possible mechanism whereby D-ribose could provide energy during strenuous exercise. It has been proposed that D-ribose could ameliorate muscle weakness by serving as an energy source [10], [11], but there is little research that specifically addresses this topic. While it is known that D-ribose is well absorbed in the small intestine [23] and that pentose sugars can be reversibly converted into hexose sugars, most sources focus on the production of ribose from glucose. Nevertheless, there is evidence in the literature to suggest that Type I skeletal fibers can catabolize AMP molecules in order to create free ribose as a potential energy source. The strongest evidence supporting the catabolism of AMP comes from observations of an “adenylate deficit” following strenuous exercise and a spike in levels of hypoxanthine in the serum after strenuous exercise [24], [25]. In brief, it is known that the total pool of adenylate molecules (i.e., ATP+ADP+AMP) in skeletal muscle tissue is significantly decreased after exercise but recovers over time. The drop in the number of adenylates coincides with a sharp increase in serum levels of hypoxanthine (a breakdown product of AMP), suggesting that excess AMP is broken down into its purine base (hypoxanthine) and phospho-ribose backbone (ribose-1P) after exercise. An excess of free ribose-1P can then be used to fuel glycolysis, since three ribose-1P molecules can be converted into two molecules of fructose-6P and one molecule of glyceraldehyde-3P via the non-oxidative phase of the pentose phosphate pathway.

After treating myositis mice for 6 weeks with daily oral supplements of D-ribose, we observed that the treated mice failed to show any improvement by any measurement. Mice with myositis fared poorly when compared to their healthy littermates, and treatment with D-ribose had no significant effect on either diseased or healthy mice. Treatment with D-ribose did not protect against muscle wasting or prevent an overall loss in body weight. In addition, D-ribose did not protect against the loss of muscle function (maximal and specific force generation) in the myositis mice. Histologically, treatment with D-ribose did not appear to have any statistically significant effect on the degree of muscle fiber damage or infiltration lymphocytes into the mouse skeletal muscle tissues. In terms of muscle fatigue, supplementation with D-ribose had no statistically significant effect on muscle fatigue in mice.

After performing QRT-PCR analysis on the genes related to the internal production of D-ribose (via AMP catabolism), we found that the first enzyme utilizing ingested D-ribose, RBKS, was expressed at very low basal levels in both healthy and myositis mice. The lack of other observed changes in gene expression suggests that the ingested D-ribose had little regulatory effect on the expression of these genes. We propose that the lack of effect of D-ribose in treated mice is due to a low basal expression of RBKS in the skeletal muscle, resulting in the utilization of an insignificant amount of the molecule. A comparison of RBKS expression profiles in humans and mice using publicly available data (NCBI UniGene Hs.11916 ) suggests that both humans and mice have low basal expression of RBKS in skeletal muscle. While we cannot directly extrapolate from this mouse model, the suggestion that D-ribose cannot be utilized by skeletal muscle tissue is in agreement with prior publications in humans showing that D-ribose has no effect on muscle function in healthy patients [15], [16], [17].

In fact, a pattern of potential harm becomes apparent after examining body weight, voluntary activity, muscle mass, and muscle function. In all of these analyses, the treated mice performed worse on average than the untreated controls. Although this drop in performance was not statistically significant in any single measured parameter, the consistency of this pattern suggests that daily oral D-ribose has potentially negative consequences. The mechanism behind this consistent drop in performance is not known. There was some prior concern that D-ribose supplements would allow greater proliferation of lymphocytes, since ribose can be a limiting factor in the synthesis of new DNA in dividing cells. However, the histology immunohistochemistry and QRT-PCR results indicate that there were no apparent differences between treated and untreated animals in the degree of lymphocyte infiltration. Similarly, QRT-PCR analysis for several inflammatory markers indicated that D-ribose showed no effect on inflammation.

In summary, the results of this research refute the hypothesis that oral supplements of D-ribose can be used to treat the muscle weakness in myositis. Contrary to the initial hypothesis, animals treated with D-ribose showed worse performance on average when compared to controls. The failure of D-ribose in treating myositis in this model does not invalidate the hypothesis that the catabolism of AMP provides substrates for glycolysis, because ingested D-ribose requires RBKS to be utilized, whereas the internal production of D-ribose via catabolism does not require RBKS. These results from this mouse model are also consistent with the broader literature on D-ribose and its lack of observed effects in humans.

## Supporting Information

Figure S1 **Immuno-histological staining for CD3 in mouse skeletal muscle showed no difference between untreated and treated animals.** Sections of quadriceps tissue were stained for CD3 via DAB deposition. Healthy mice showed negative staining regardless of treatment (*A&B*). Untreated myositis mice (HT) were positive for foci of CD3+ cells between fibers (*C*), as were HT mice treated D-ribose (*D*). Treatment with D-ribose did not significantly decrease the number of CD3+ foci per cross-section (*E*).
(TIF)
