# Supplementary figures and images for: Daily Supplementation of D-ribose Shows No Therapeutic Benefits in the MHC-I Transgenic Mouse Model of Inflammatory Myositis
Source: PLoS One. 2013 Jun 13;8(6):e65970. doi: 10.1371/journal.pone.0065970 (PMC3681851; doi:10.1371/journal.pone.0065970)

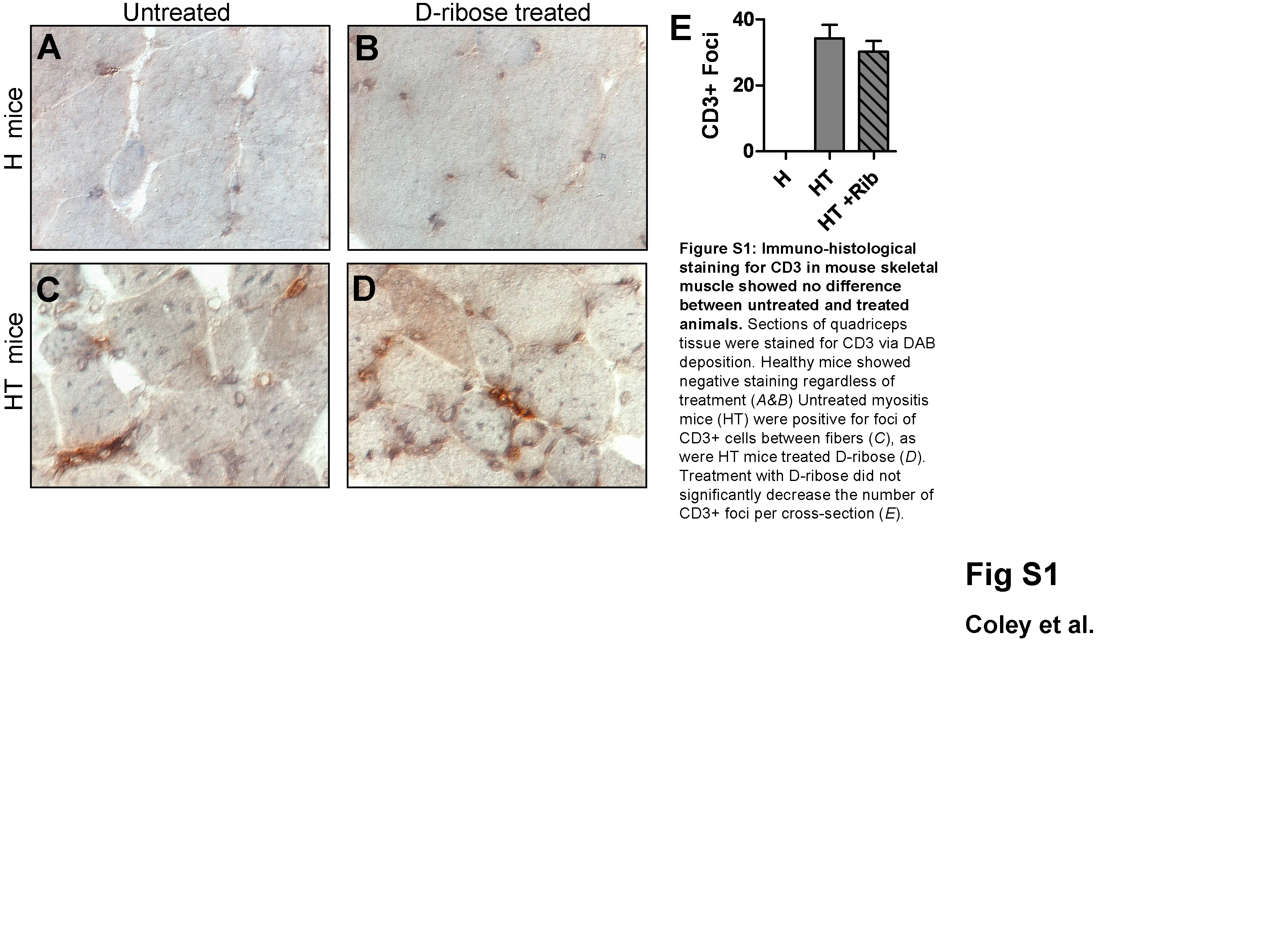

Supplement: Figure S1 — Immuno-histological staining for CD3 in mouse skeletal muscle showed no difference between untreated and treated animals. Sections of quadriceps tissue were stained for CD3 via DAB deposition. Healthy mice showed negative staining regardless of treatment (A&B). Untreated myositis mice (HT) were positive for foci of CD3+ cells between fibers (C), as were HT mice treated D-ribose (D). Treatment with D-ribose did not significantly decrease the number of CD3+ foci per cross-section (E). (TIF) [file pone.0065970.s001.tif]
